# Influence of Infrared Thermography Predictors in Dental Implant Osteotomies: An Ex Vivo Study

**DOI:** 10.1016/j.identj.2025.03.009

**Published:** 2025-03-30

**Authors:** Gabriel Barriga-Yauri, Daniel Alvitez-Temoche, Franco Mauricio, Fran Espinoza-Carhuancho, Ivan Calderon, Julia Medina, Frank Mayta-Tovalino

**Affiliations:** a Vicerrectorado de Investigación, Research, Innovation and Entrepreneurship Unit, Universidad Nacional Federico Villarreal, Lima, Perú; b Bibliometrics Evidence Evaluation and Systematic Reviews Group (BEERS) Human Medicine Career, Universidad Científica del Sur, Lima, Perú; c Academic Department of Stomatology and Medical Surgery, Faculty of Dentistry, Universidad Nacional Mayor de San Marcos, Lima, Perú

**Keywords:** Dental implant, Ex vivo study, Infrared, Thermographic alterations

## Abstract

**Objective:**

To evaluated the contribution of irrigation, drill type, motor, and dental implant system on infrared thermographic variations in osteotomies.

**Methods:**

Osteotomy sites for 240 implants were prepared with three implant systems (Arcys, NeoBiotech and Osstem), with 80 samples analyzed in each group. Each group was further subdivided according to the drilling conditions (with and without irrigation) and motor type (Coxo, W&H, Dentflex, Driller). The drill sequence included baseline, pilot, second and third drills at 1200 rpm and 40 Ncm. Infrared thermographic measurements were performed using a Fluke TiS55+ camera, with a resolution of 220 × 165 pixels and a temperature range of −20 °C to 450 °C. Statistical analysis consisted of ANOVA with Bonferroni post hoc test and linear regression model, with a view to evaluate the influence of the variables on final temperature changes.

**Results:**

Irrigation caused a much lower temperature across all implant systems (*P* < .001). When not irrigated, Arcys showed the highest temperatures, while in both NeoBiotech and Osstem the heat dissipated better. The Bonferroni post hoc test revealed no significant temperature difference existed among implant systems without irrigation. With irrigation, however, Arcys had a higher temperature than NeoBiotech and Osstem (*P* < .001). The type of motor had no statistically significant influence on the temperature of the final drilling (*P* > .05). Based on the regression analysis, the baseline, pilot, and second drill temperatures were the strongest predictors of the final drill temperature, with irrigation influencing the effects of the drill temperature.

**Conclusion:**

Irrigation decreased the thermal stress during osteotomy, while NeoBiotech and Osstem showed better heat dispersing abilities. The motor type does not have a significant influence on the temperature differences. These findings establish the basic need for effective irrigation protocols to avoid thermal osteodisruption and promote osseointegration in implantology.

## Introduction

In recent decades, significant progress has been made in the diagnosis and management of oral diseases, but tooth loss due to disease or injury remains a common problem.^1^ Dental implants have established themselves as the preferred option for replacing missing teeth, thanks to their high efficacy and success rate.^2^ Most practitioners consider a gentle, trauma-free surgical procedure to be critical in predicting the long-term success of dental implants. This is because any mechanical or thermal damage during site preparation may cause bone necrosis and compromise osseointegration.^3^

Factors influencing the osteotomy process include bone quality, surgical technique used, drill design and condition, irrigation methods, materials used, and parameters such as time, loading and speed during the procedure.^4^ According to the condition of the drills, the wear is due to continuous use for osteotomies, reducing their cutting capacity and increasing heat generation. For this reason, it is appropriate to properly use the correct implant systems and sterilizers, taking into account that there are studies where it is suggested that aseptic techniques could affect the success of the implant.^5^

Infrared thermography (IRT) is a noninvasive method that transcends traditional visual limitations by analyzing local blood flow through the accurate detection of variations in skin temperature between different areas of the body.^6^^,^^7^ This tool can anticipate potentially necrotic areas in compressed tissues and detect signs of chronic wound infection before they become clinically clear. It is especially useful for identifying subtle thermal changes that may indicate the onset of complications, which is crucial for early intervention to prevent more serious damage.^8^ In addition, IRT captures and visualizes the thermal energy of an object, creating images that show how temperature is distributed across its surface, making it especially useful for detecting changes.^9^

In the area of dentistry, IRT has been applied in different specialties where it is used to measure thermal variations in bone tissue.^9^ It has also been used for the diagnosis and treatment of various dental medical conditions, including dental pulp pathologies, inflammatory arthritis, and temporomandibular joint disorders.^10^ The application of IRT in dental medicine has proven to be a complementary and valuable tool for both diagnosis and treatment because it accurately detects thermal changes in orofacial structures, which is essential for characterizing and monitoring various dental and maxillofacial conditions.^11^ The wide range of applications of IRT underscores its tremendous capabilities; however, to achieve more widespread adoption, increased investments in areas such as automation and strengthening its reproducibility and objectivity are critical.^12^

Therefore, the aim of this study was to evaluate the influence of infrared thermography predictors on dental implant osteotomies: an ex vivo study.

## Materials and methods

### Ethics

The present study had no ethical implications because it was an ex vivo design in which samples of cow ribs obtained from animals that had already been slaughtered for human consumption were used; thus, no type of intervention was performed on the animals for the purposes of this research. Special emphasis was placed on applying and respecting all the existing norms and recommendations that guide the use of animal tissues in ex vivo studies of this type. In any case, the approval and opinion of the Ethics Committee of the Faculty of Dentistry of the Federico Villarreal National University of Lima (Peru) was requested (Code N°032-02-2025).

### Study design

This study was carried out in an ex vivo model with the aim of evaluating the impact of irrigation, type of drill, type of motor and dental implant system on thermal alterations, measured by infrared thermography, during osteotomy procedures. The CRIS Guidelines (Checklist for Reporting in vitro Studies) were used for data reporting.^13^

### Sample size

In total, N = 240 bone surgical beds were used, distributed in three groups according to the implant system (Arcys, NeoBiotech, Osstem) with 80 samples per group, according to the type of motor to be used with the samples for each group. Each group was divided into subgroups based on the drilling conditions, i.e., irrigated and non-irrigated, based on the motor type (Coxo, W&H, Dentflex, Driller). The sample size was established from the collection of data from a pilot study, with a power analysis of the pilot study to make it feasible to have adequate statistical power (α = 0.05, β = 0.80).

### Selection criteria

Cow rib samples were used, as they most closely resembled human bones in density and in their response to drilling procedures. Only intact bone samples, free of fractures and pathology, were accepted. All bone samples were kept at 4 °C until the time of testing. Bone samples were excluded if they showed visible characteristics of a fate that had previously caused different types of injury or deformation, and also if they had been exposed to previous environmental conditions that could be seen to have altered their thermal properties (e.g., freezing or long periods of time in environmental conditions that could have affected them).

### Obtaining the beef ribs

The beef ribs used in this study were acquired from the Frigorífico Yerbateros slaughterhouse, located in the city of Lima, Peru (https://frigorificoyerbateros.pe/). The ribs were randomly selected, ensuring that all ribs were of similar size and condition to preserve homogeneity in the results of this study.

### Preparation and conditioning

The cow ribs were obtained, after which the samples were cleaned and conditioned with a 10% solution of sodium hypochlorite, for which the remains of meat, fat and connective tissue were removed with the help of sterile surgical tools, in order to achieve a completely clean bone surface. Then, the ribs were subjected to a superficial cleaning with chlorhexidine 0.12% in order to avoid contamination that could modify the parameters of interest and were kept at −20 °C under controlled temperature and humidity conditions so that decomposition would not take place.

### Drilling protocol

Drilling preparation was carried out using three types of dental implant systems such as Arcys, NeoBiotech, and Osstem, with a controlled speed of 1200 rpm and constant torque of 40 Ncm in each of the drilling stages to which the samples were subjected, which included four drilling stages: the baseline (Baseline °C), the first drill (Pilot Drill °C), the drill with the largest diameter (Second Drill °C), and the final drill (Third Drill °C). Each of the systems was evaluated under milling conditions with and without irrigation to study the effect of irrigation on the temperatures generated.

### Thermographic-infrared changes

The temperature was measured during milling using a Fluke TiS55+ thermographic camera (Washington, United States) that showed in real time the temperature response during the whole process of this technique. The camera had dimensions of 220 × 165 (36,300 pixels) and a depth of field of 353:1, generating images of the dirt in the region of interest. During milling, the camera was adjusted to generate quality images, and all measurements were consistently collected at all stages of this technique to obtain precision data regarding the thermal variations of the bone samples (Figure).

**Fig:**
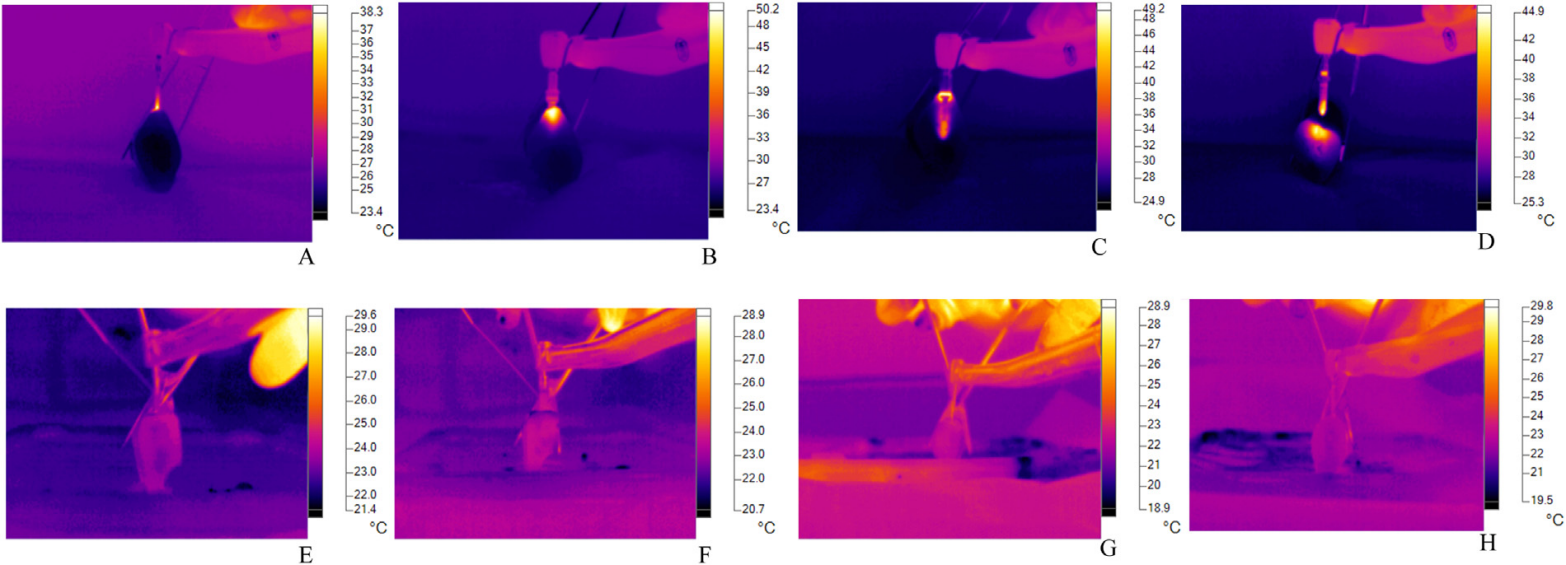
Thermographic-infrared changes (°C) with and without irrigation. A and B, Generation of progressive thermographic-infrared changes without irrigation. E-H, Thermographic-infrared changes controlled by the irrigation process.

### Analysis plan

The means and standard deviations of the continuous variables were obtained. Subsequently, their normal distribution was determined through the Shapiro–Wilk test. The dependent variable was the temperature recorded during the final drilling (Third Drill °C). To determine the effect of irrigation, drill type, motor type, and implant system on temperature, an analysis of variance (ANOVA) with the corresponding Bonferroni post hoc test was performed for each comparison between the groups. In addition, linear regression analyses were performed to test the relationship between the baseline temperature, pilot drill temperature, second drill temperature, and final temperature in each condition. All statistical analyses were performed using the Stata software, version 17.0. For all contrasts, a significance level of *P* < .05 was set.

### Results

The mean temperatures recorded in the Arcsys system without irrigation reached 26.74 ± 0.66 °C during the Third Drill and decreased further to 22.64 ± 0.77 °C with irrigation. During the Pilot Drill, this temperature was recorded as 24.94 ± 0.29 °C and 23.74 ± 0.76 °C, respectively. Statistical analysis showed highly significant temperature reduction with irrigation (*P* < .001), thus marking the role of irrigation in dissipating the heat generated in the course of performing osteotomy. (Table 1)

**Table 1:** Comparison of thermographic-infrared changes during surgical osteotomy.

		Baseline				Pilot drill °C				Second drill °C				Third drill °C				
		Without irrigation		With irrigation		Without irrigation		With irrigation		Without irrigation		With irrigation		Without irrigation		With irrigation		P*
		Mean	SD	Mean	SD	Mean	SD	Mean	SD	Mean	SD	Mean	SD	Mean	SD	Mean	SD	
	Arcys‡	22.26	0.42	21.8	0.84	24.94	0.29	23.74	0.76	25.82	0.49	22.99	0.61	26.74	0.66	22.64	0.77	>.05
Coxo	NeoBiotech†	23.37	0.13	20.46	0.12	23.86	0.12	22.47	0.13	25.87	0.13	22.77	0.13	26.57	0.13	22.37	0.13	
	Osstem§	23.75	0.10	21.55	0.10	23.95	0.10	20.25	0.10	24.15	0.10	21.25	0.10	23.35	0.10	21.15	0.10	
	*P*^⁎⁎^	.001				.001				.001				.001				
	Arcys‡	24.65	0.30	23.59	0.26	25.28	0.31	24.29	0.72	25.91	0.27	24.76	0.87	25.8	0.37	23.93	0.90	>.05
W&H	NeoBiotech†	22.95	0.10	21.43	0.09	23.72	0.10	21.35	0.10	23.42	0.91	22.31	0.09	25.12	0.10	21.62	0.10	
	Osstem§	23.45	0.10	20.75	0.10	24.45	0.10	20.85	0.10	24.75	0.10	20.65	0.10	24.45	0.10	21.45	0.10	
	*P*^⁎⁎^	.001				.001				.001				.001				
	Arcys‡	24.45	0.15	22.75	0.15	26.05	0.15	26.05	0.15	25.86	0.15	21.85	0.15	24.45	0.15	22.96	0.15	>.05
Dentflex	NeoBiotech†	21.62	0.13	21.65	0.10	22.65	0.31	21.38	0.15	22.29	0.17	21.55	0.10	23.05	0.10	21.23	0.11	
	Osstem§	23.55	0.10	21.45	0.10	24.15	0.10	22.05	0.10	29.15	0.10	22.65	0.10	27.65	0.10	21.25	0.10	
	*P*^⁎⁎^	.001				.001				.001				.001				
	Arcys‡	22.73	0.17	21.75	0.15	24.25	0.15	21.55	0.15	24.71	0.16	21.25	0.15	24.65	0.15	21.35	0.15	>.05
Baby driller	NeoBiotech†	23.07	0.13	21.57	0.13	24.97	0.13	21.87	0.13	26.37	0.13	22.27	0.13	27.57	0.13	21.37	0.13	
	Osstem§	21.45	0.10	22.25	0.10	21.15	0.10	21.65	0.10	25.35	0.10	21.85	0.10	24.45	0.10	23.55	0.10	
	*P*^⁎⁎^	.001				.001				.001				.001				

P* Shapiro Wilk Normality Test

*P*^⁎⁎^ Student t test (Between with and without irrigation)

‡ Arcsys: Titanium nitride coated drills, the drilling sequence was pilot drill Ø2.4mm, the next drill Ø 2.9mm, and 3.4mm.

† NeoBiotech: The initial drill is called Lindermann Ø2mm, Ø2.2mm and Ø3.0mm.

§ Osstem: The initial drill is called LanceDrill, Ø2.2mm and Ø3.0mm.

In the NeoBiotech system, the temperatures recorded without irrigation ranged from 23.86 ± 0.12 °C during the Pilot Drill, to 25.87 ± 0 .13 °C during the Second Drill, and up to 26.57 ± 0.13 °C during the Third Drill. With irrigation, the recorded temperatures dropped down to 22.47 ± 0.13 °C, 22.77 ± 0.13 °C, and 22.37 ± 0.13 °C, respectively. The statistical analysis showed a highly significant difference between ip conditions with and without irrigation (*P* < .001), corroborating the efficacy of irrigation in reducing thermal stress upon bone tissue. (Table 1)

The Osstem system reported the highest temperatures without irrigation, with values measuring 29.15 ± 0.10 °C during the Second Drill and 27.65 ± 0.10 °C during the Third Drill. Irrigation resulted in highly significant decreased temperatures to 22.65 ± 0.10 °C and 21.25 ± 0.10 °C, for each drill step. During the Pilot Drill, irrigation reports at 23.95 ± 0.10 °C with no irrigation and 20.25 ± 0.10 °C with irrigation. Statistical analysis confirmed that during all stages of drilling, irrigation produced a significant decrease in temperature (*P* < .001), highlighting its importance in near-elimination of excessive heat rising through the bone. From the performed analyses in every system, it was found that irrigation significantly reduced the temperature (*P* < .001), which is an important factor in avoiding possible thermal injuries to bone tissue during an osteotomy. (Table 1)

The Bonferroni post hoc analysis for Third Drill °C by Implant Type revealed that, in the absence of irrigation, there were no statistically significant differences in temperature between implant systems (*P* > .05). However, under irrigated conditions, Arcys had significantly higher temperatures than both NeoBiotech (*P* < .001) and Osstem (*P* < .001), while no significant differences were found between NeoBiotech and Osstem (*P =* .605). These findings suggest that irrigation is a significant factor in differentiating thermal performance among implant systems, with NeoBiotech and Osstem dissipating heat better than Arcys. (Table 2)

**Table 2:** Bonferroni multiple comparisons for the third drill °C by implant type.

	Comparison (Implants)	Mean Difference	95% CI	Adjusted *P* value
Third drill °C without irrigation	Arcys vs NeoBiotech	0.14	(−0.75, 1.04)	.907
	Arcys vs Osstem	−0.45	(−1.45, 0.54)	.398
	NeoBiotech vs Osstem	−0.60	(−1.58, 0.37)	.200
Third drill °C with irrigation	Arcys vs NeoBiotech	−1.07	(−1.60, −0.54)	<.001
	Arcys vs Osstem	−0.87	(−1.40, −0.34)	<.001
	NeoBiotech vs Osstem	0.20	(−0.32, 0.72)	.605

The Bonferroni post hoc analysis for Third Drill °C with respect to Motor Type indicated that there were no statistically significant differences in temperature between the motor systems, regardless of irrigation (*P* > .05). Without irrigation, the highest mean difference was Coxo vs Dentflex (−0.50 °C, *P =* .642), while with irrigation, the largest difference was W&H vs Dentflex (−0.52, *P =* .264). Neither was statistically significant. It suggests that the motor type did not influence osteotomy temperature significantly in the presence or absence of irrigation. (Table 3)

**Table 3:** Bonferroni multiple comparisons for the third drill °C by motor type.

	Comparison (motors)	Mean difference	95% CI	Adjusted *P* value
Third drill °C without irrigation	Coxo vs W&H	−0.40	(−1.42, 0.61)	.782
	Coxo vs Dentflex	−0.50	(−1.52, 0.51)	.642
	Coxo vs Driller	0.00	(−1.01, 1.02)	1.000
	W&H vs Dentflex	−0.10	(−1.11, 0.91)	.996
	W&H vs Driller	0.40	(−0.60, 1.41)	.778
	Dentflex vs Driller	0.50	(−0.49, 1.50)	.637
Third drill °C with irrigation	Coxo vs W&H	0.28	(−0.63, 1.19)	.761
	Coxo vs Dentflex	−0.24	(−1.15, 0.67)	.835
	Coxo vs Driller	0.03	(−0.87, 0.94)	.999
	W&H vs Dentflex	−0.52	(−1.43, 0.39)	.264
	W&H vs Driller	−0.24	(−1.15, 0.66)	.830
	Dentflex vs Driller	0.27	(−0.63, 1.19)	.768

For each degree that the baseline temperature without irrigation (Baseline °C) increases, the final drill temperature (Third Drill °C) decreases by 1.08 °C (95% CI: −1.24 to −0.92). This is statistically significant. For each degree that the temperature increases with the Pilot Drill without irrigation, the temperature of the final drill (Third Drill °C) increases by 0.95 °C (95% CI: 0.78-1.12), which is statistically significant. Similarly, for each degree that the temperature increases with the Second Drill without irrigation, the temperature of the final drill (Third Drill °C) increases by 0.57 °C (95% CI: 0.49-0.64), which is statistically significant. (Table 4)

**TABLE 4:** Linear regression analysis for the third drill °C without irrigation.

	Third drill °C
Variables	β (95% CI)
Baseline °C without irrigation	−1.08 (95% CI: −1.24 to −0.92)
Pilot drill °C without irrigation	0.95 (95% CI: 0.78-1.12)
Second drill °C without irrigation	0.57 (95% CI: 0.49-0.64)
Baseline °C with irrigation	−0.73 (95% CI: −0.87 to −0.60)
Pilot drill °C with irrigation	−0.42 (95% CI: −0.64 to −0.20)
Second drill °C with irrigation	1.14 (95% CI: 1.01-1.27)
Third drill °C with irrigation	0.14 (95% CI: −0.01 to 0.29)
Motor type	0.12 (95% CI: 0.04-0.20)
Implant system	0.07 (95% CI: −0.14 to 0.28)

When irrigation is applied, for each degree that the baseline temperature with irrigation (Baseline °C) increases, the final drill temperature (Third Drill °C) decreases by 0.73 °C (95% CI: −0.87 to −0.60), which is statistically significant. For each degree that the temperature increases with the Pilot Drill with irrigation, the temperature of the final drill (Third Drill °C) decreases by 0.42 °C (95% CI: −0.64 to −0.20), which is statistically significant. Conversely, for each degree that the temperature increases with the Second Drill with irrigation, the temperature of the final drill (Third Drill °C) increases by 1.14 °C (95% CI: 1.01-1.27), which is statistically significant. (Table 4)

The Motor type variable showed that for each degree increase in motor temperature, the final drill temperature (Third Drill °C) increased by 0.12 °C (95% CI: 0.04-0.20), which is statistically significant. The Implant system had no significant impact on the final drill temperature (Third Drill °C) (β = 0.07, 95% CI: −0.14 to 0.28).

For each degree that the baseline temperature without irrigation (Baseline °C) increases, the final drill temperature (Third Drill °C) increases by 0.70 °C (95% CI: 0.41-0.99), which is statistically significant. However, for each degree that the temperature increases with the Pilot Drill without irrigation, the temperature of the final drill (Third Drill °C) decreases by 0.78 °C (95% CI: −1.05 to −0.52), which is statistically significant. Similarly, for each degree that the temperature increases with the Second Drill without irrigation, the final drill temperature (Third Drill °C) decreases by 0.21 °C (95% CI: −0.37 to −0.05), which is statistically significant. Finally, for each degree that the temperature increases with the Third Drill without irrigation, the temperature of the final drill (Third Drill °C) increases by 0.22 °C (95% CI: −0.01 to 0.45), but this result is not statistically significant (*P* > .05). (Table 5)

**Table 5:** Linear regression analysis for the third drill °C with irrigation.

	Third drill °C
Variables	β (95% CI)
Baseline °C without irrigation	0.70 (95% CI: 0.41-0.99)
Pilot drill °C without irrigation	−0.78 (95% CI: −1.05 to −0.52)
Second drill °C without irrigation	−0.21 (95% CI: −0.37 to −0.05)
Third drill °C without irrigation	0.22 (95% CI: −0.01 to 0.45)
Baseline °C with irrigation	0.42 (95% CI: 0.20-0.65)
Pilot drill °C with irrigation	0.98 (95% CI: 0.76-1.21)
Second drill °C with irrigation	−0.43 (95% CI: −0.73 to −0.13)
Motor type	−0.03 (95% CI: −0.13 to 0.08)
Implant system	0.17 (95% CI: −0.09 to 0.43)

When irrigation is applied, for each degree that the baseline temperature with irrigation (Baseline °C) increases, the final drill temperature (Third Drill °C) increases by 0.42 °C (95% CI: 0.20-0.65), which is statistically significant. For each degree that the temperature increases with the Pilot Drill with irrigation, the temperature of the final drill (Third Drill °C) increases by 0.98 °C (95% CI: 0.76-1.21), which is statistically significant. However, for each degree that the temperature increases with the Second Drill with irrigation, the final drill temperature (Third Drill °C) decreases by 0.43 °C (95% CI: −0.73 to −0.13), which is statistically significant. (Table 5)

The Motor Type variable had no significant effect on the final drill temperature (Third Drill °C) (β = −0.03, 95% CI: −0.13 to 0.08), while the Implant System had a slight positive effect (β = 0.17, 95% CI: −0.09 to 0.43), but this was also not statistically significant. (Table 5)

## Discussion

Remarkable results were obtained, which clearly show that irrigation is a very effective way to keep the temperature low during all phases of drilling. This is important because it helps to protect the bone tissue from heat damage. It was also found that the type of motor was not a determining factor in the final temperature of the drill, suggesting that the motor system does not exert significant control over the heat produced during osteotomy. The regression analysis that was performed showed that, without irrigation, the reference, pilot and second drill temperatures were the most important factors in predicting the final drill temperature. On the other hand, with irrigation, the reference and pilot burr temperatures showed a negative correlation with the final burr temperature. The shape of the burs should be considered since they influence the heat produced; a bad design, such as a lack of angle or a small angle of amplitude, can overheat the bone.^14^^,^^15^

For example, a study by Kosior et al analyzed the amount of heat produced by implant systems (Straumann, Osstem and AnyRidge) when preparing the site in the bone for placement. It evaluated factors such as bone density, the design of the drill used, the depth and diameter of drilling, and the rotational speed of the drill. It also examined how cooling and drill speed influence the temperature during the implant bed preparation. The study found that the ideal speed for preparing the implant site is 1200 rpm, which matches the speed used in our research. This is because, when using a lower speed of 800 rpm, more time is required to drill, which increases friction and, therefore, heat. On the other hand, by increasing the speed to 1500 rpm, the excessive rotation also raises the friction and temperature.^16^

A noteworthy result of the above study was the maximum temperature (50.8 °C) recorded for the AnyRidge pilot drill at 1500 rpm without cooling as opposed to our study in which the Osstem system reported the highest temperatures without irrigation, with values of 29.15 ± 0.10 °C during the second simulation and 27.65 ± 0.10 °C during the third simulation performed.16 Although this study was carried out with pork ribs and ours with bovine ribs, the veracity of the information obtained is reliable due to the fact that although it does not totally reflect the human bone model, there is the possibility of obtaining very similar results.^17^

Another study by Kapse et al^18^ aimed to evaluate different factors associated with implant drills causing heat formation and temperature increase in the course of osteotomy obtained as an outstanding result, the temperature change at the drilling site with saline solution at 5 °C was 24.47 ± 0,.8 and 24.82 ± 0.080 and at 25 °C was 29.27 ± 1.77 and 29.7 ± 1.85 for Groups A and B that were formed in that study. The study also included room temperature, which had no statistically significant differential effect on the final result and could be used to justify the use of this variable. What did have a difference was in the use of irrigant, which agrees with our results, where the decrease in temperature is highlighted and allows for its control.^18^

On the other hand, Raj et al^19^ Performed an analysis of the factors that determine the thermal changes at the osteotomy site. Among the main components, he used the bovine femur, a drilling machine (COXO Dental Physiodispenser, INDIA) and two drills (ADIN Dental Implant Systems Ltd., Israel). He used different speeds than ours such as 2000 and 2500 rpm, but the results did not indicate a heat generated greater than 47 ° to cause bone necrosis. He also highlighted the use of irrigation, which indicated that it helped to control and improve the osteotomy temperature. The authors indicate that implant failures can be caused by a combination of multiple factors and not always by bone necrosis, but bone necrosis is still the major cause of dental implant failures.^19^

The implementation of IRT in our study was fundamental to achieve accurate results, as it acts as an effective predictor. This is because IRT represents an advanced and complete technological solution at present. Its remarkable sensitivity to temperature changes allows its instruments to identify even the slightest temperature variations using thermal imaging and temperature gradients that are visualized through color coding.^20^

The present research had the remarkable strength of analyzing several variables, which not only enriched the statistical data but also greatly increased the quality of the results. The plurality of variables provides a global and detailed view that allows a more comprehensive understanding of the various factors that determine thermographic variations during osteotomy. However, this research has certain important limitations. First, the use of bovine ribs as a bone model, although it simulates human bone, does not reproduce it. This difference implies a caveat that could mean that the results cannot be extrapolated to clinical practice in humans. Second, the experimental environment, especially the ambient temperature, is an important factor in the results. Variations in ambient temperature mean that thermographic measurements may differ, and with it, the interpretation of the data. All these limitations suggest that further studies should be performed using a bone model more representative of the human bone and rigorously controlling environmental conditions; only through replication of studies under similar conditions can greater consistency and validity in the results produced be obtained.

## Conclusions

This study evaluated the effect on the temperature of irrigation, implant systems, and motor types during osteotomy procedures. These results underscore that irrigation is highly effective at minimizing the temperature at all three drilling stages, thereby lessening the thermal stress on the bone tissue. In all implant systems (Arcys, NeoBiotech, and Osstem), temperatures were significantly reduced when irrigation was performed (*P* < .001), with Arcys exhibiting the highest temperatures without irrigation, with NeoBiotech and Osstem better at heat dissipation when irrigation was performed, particularly under irrigated conditions. The type of motor did not significantly influence the final drill temperature (*P* > .05), indicating that the motor system exerts no significant influence on the thermal performance during osteotomy. Results were supported by post hoc Bonferroni analysis, showing no temperature differences among the motor systems, associated with or without irrigation. Regression analysis revealed that baseline, pilot, and second drill temperatures were among the more important parameters in predicting the final drill temperature, predominantly under nonirrigated conditions. Conversely, it was found that under irrigation baseline and pilot drill temperatures displayed a negative correlation with the final drill temperature; in this case, the implant system exerted no meaningful effect (β = 0.07), while the motor type had a slight positive effect (β = 0.12). Overall, irrigation appears to be the prime operator in temperature control and the prevention of thermal bone damage during osteotomy procedures.

## Conflict of interest

None disclosed.
